# Crystal structure of a new polymorph of 3-acetyl-8-meth­oxy-2*H*-chromen-2-one

**DOI:** 10.1107/S2056989019015159

**Published:** 2019-11-15

**Authors:** Gabino Gonzalez-Carrillo, Roberto Muñiz-Valencia, Efren V. García-Báez, Francisco J. Martínez-Martínez

**Affiliations:** a Facultad de Ciencias Químicas, Universidad de Colima, km 9 carretera, Colima-Coquimatlán, 28400, Coquimatlán, Colima, Mexico; b Laboratorio de Quimica Supramolecular y Nanociencias, Unidad Profesional, Interdisciplinaria de Biotecnología, Instituto Politécnico Nacional, Avenida Acueducto s/n, Barrio La Laguna Ticomán, Cd. de Mexico 07340, Mexico

**Keywords:** crystal structure, polymorph, hydrogen bond, coumarin, 3-acetyl-8-meth­oxy-coumarin

## Abstract

A new polymorphic form of the title compound, C_12_H_10_O_4_, is described in the ortho­rhom­bic space group *Pbca* and *Z* = 8, as compared to polymorph I, which crystallizes in the monoclinic space group *C*2/*c* and *Z* = 8 [Li *et al.* (2012). *Chin. J. Struct. Chem.* **31**, 1003–1007.]. In polymorph II, the coumarin ring system is almost planar (r.m.s. deviation = 0.00129 Å). In the crystal, mol­ecules are connected by C*sp* ^3^—H⋯O and C_ar_—H⋯O hydrogen bonds, forming mol­ecular sheets linked into zigzag shaped layers along the *b*-axis direction. The three-dimensional lattice is assembled through stacking of the zigzag layers by π–π inter­actions with a centroid-to-centroid distance of 3.600 (9) Å and anti­parallel C=O⋯C=O inter­actions with a distance of 3.1986 (17) Å, which give rise to a helical supra­molecular architecture.

## Abstract 2

A new polymorphic form of 3-acetyl-8-meth­oxy-2*H*-chromen-2-one is described and compared with the previously reported polymorph. In the crystal, hydrogen bonds, π–π inter­actions and anti­parallel C=O⋯C=O inter­actions give rise to a helical supra­molecular architecture

## Chemical context   

Derivatives of 2*H*-chromen-2-one are some of the most important heterocycles in natural and synthetic organic chemistry. These substances are bioactive compounds and have a wide range of applications in the medical field (Gaudino *et al.*, 2016 ▸) showing, for example, anti-HIV, anti­mutagenic, anti­cancer and anti­tumor activities among others (Vekariya & Patel, 2014 ▸). They are synthesized using classical methodologies such as the Pechmann or Knoevenagel reactions, as well as recent methodologies such as the metathesis cyclization (Salem *et al.*, 2018 ▸) or alkynoates cyclization (Liu *et al.*, 2018 ▸).

The disposition of the crystalline lattices of coumarin derivatives is driven by a great variety of inter­molecular inter­actions (Santos-Contreras *et al.*, 2009 ▸). This working group has reported the participation of π–π stacking inter­actions, hydrogen-bonding and dipole–dipole inter­actions involving the carbonyl group (Gómez-Castro *et al.*, 2014 ▸) in the determination of the 1D, 2D and 3D supra­molecular assemblies of crystalline structures for different compounds (González-Padilla *et al.*, 2014 ▸). This report describes the structure of a second polymorph of the title compound and the importance of C—H⋯O, C=O⋯C=O and π–π stacking inter­molecular inter­actions in crystal packing.

## Structural commentary   

**Figure 1:**
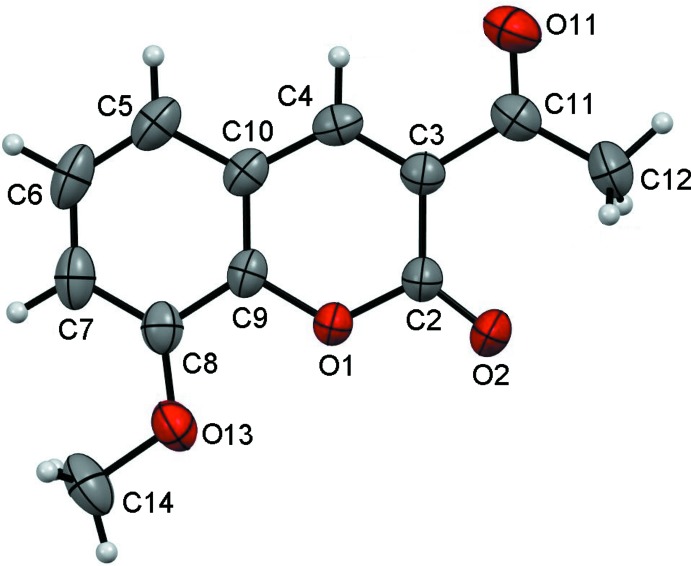
*ORTEP* plot of polymorph II of the title compound with the atom-numbering scheme. Displacement ellipsoids are drawn at the 50% probability level.

The title polymorph II (Fig. 1 ▸) crystallizes in the ortho­rhom­bic system, space group *Pbca*, with eight mol­ecules in the unit cell whereas polymorph I (Li *et al.*, 2012 ▸) crystallizes in the monoclinic system in space group *C*2/*c*, also with eight mol­ecules in the unit cell. In polymorph II, the coumarin skeleton is almost planar (r.m.s. deviation = 0.00129 Å) with dihedral angles O1—C9—C10—C5 and C8—C9—C10—C4 of 179.20 (10) and 179.87 (11)°, respectively. In contrast, in polymorph I the benzene and lactone rings deviate slightly from planarity by 2.76 (3)°. The acetyl and meth­oxy groups of polymorph II are almost coplanar with the coumarin ring, with torsion angles C2—C3—C11—C12 = −1.25 (18)° and C14—O13—C8—C7 = −2.70 (18)°.
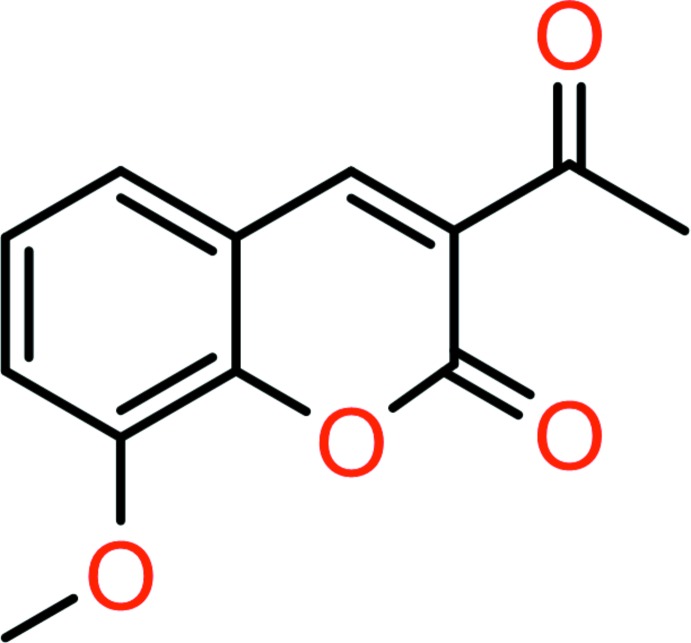


## Supra­molecular features   

**Figure 2:**
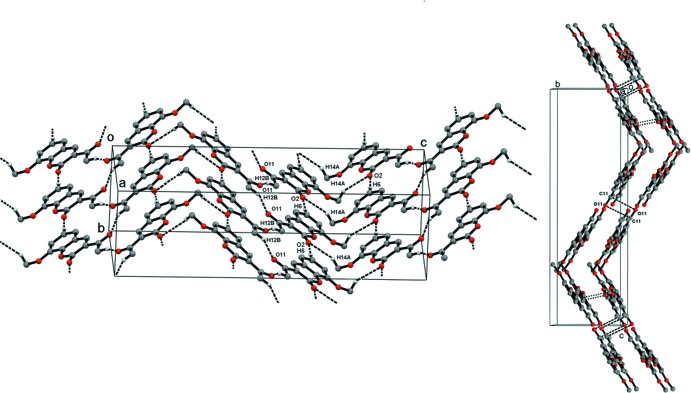
Packing of mol­ecules in polymorph II by C—H⋯O hydrogen bonding and the packing of parallel sheets connected *via* C=O⋯C=O and weak π–π inter­actions. Dotted lines depict the inter­molecular inter­actions.

The crystal network of the title compound (polymorph II) is assembled by zigzag shaped mol­ecular layers that extend approximately in the (012) and (01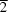) planes, forming an angle of 116.2°. In the flat section of the zigzag layer 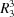
(18) motifs are formed by C6—H6⋯O2^ii^ and C12—H12*B*⋯O11^i^ hydrogen bonds (Table 1 ▸). These inter­molecular inter­actions impart stability to the 2D sheet, while weak C14—H14*A*⋯O2^iii^ inter­actions generate an 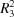
(16) motif at the inter­section of the planes (Fig. 2 ▸). Adjacent layers, separated by a distance of 3.4083 (5) Å, are connected by π–π stacking inter­actions with a centroid-to-centroid distance of 3.600 (9) Å and a slippage of 1.160 Å. In addition to the π stacking, layers are stabilized by anti­parallel C=O⋯C=O inter­actions (Allen *et al.*, 1998 ▸) involving the acetyl group separated by a distance of 3.1986 (17) Å.

**Figure 3:**
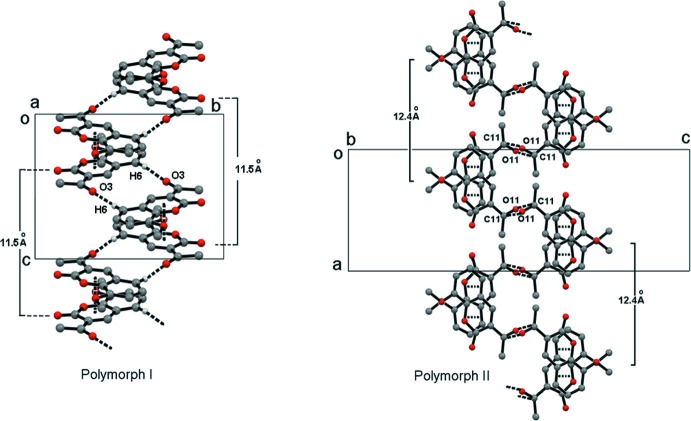
Helical conformation in the packing of polymorphs I and II of 3-acetyl-8-meth­oxy-2*H*-chromen-2-one. Dotted lines depict inter­molecular inter­actions.

The supra­molecular array of polymorph II exhibits a helical conformation, like polymorph I (Li *et al.*, 2012 ▸). However, in polymorph II the C=O⋯C=O inter­actions form the central axis of the helix whilst π–π inter­actions between the aromatic and lactone rings, aligned in a head-to-tail conformation, control the rotation of the structure. In polymorph I, the helical axis is built by hydrogen bonds and face-to-face π–π stacking inter­actions of the benzofused rings (Fig. 3 ▸). For polymorph I, a complete rotation of the helix is performed in 11.5 Å, while in polymorph II the displacement of the helix in a whole rotation is 12.4 Å.

## Hirshfeld surface and 2D fingerprint plots   

**Figure 4:**
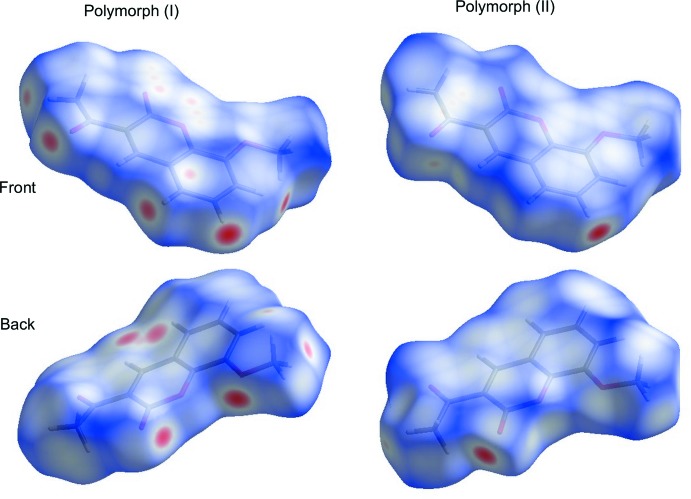
Hirshfeld surfaces for polymorphs I and II showing both sides of the mol­ecules. Red areas represent contacts shorter than the sum of the van der Waals radii, blue areas represent zones where the shortest distance between atoms is larger than the sum of van der Waals radii and white areas are zones close to the sum of van der Waals radii.

**Figure 5:**
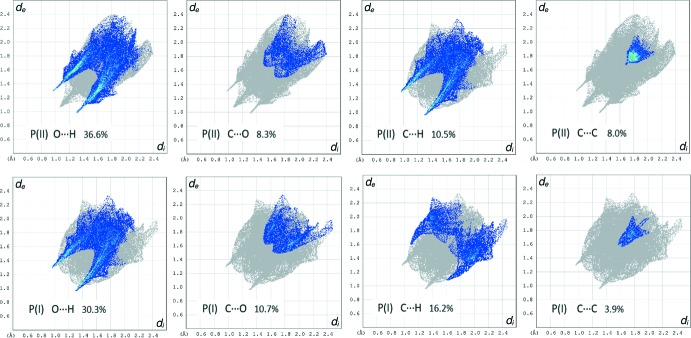
Comparison of several inter­molecular inter­actions (blue areas) involved in the crystal packing of polymorphs I and II by decomposition of two-dimensional fingerprint plots. Green areas represent a greater abundance of close contacts and the full fingerprint appears beneath each decomposed plot as a grey shadow.

In order to better understand the crystal packing of both polymorphs, Hirshfeld surface analyses and 2D fingerprint plots were carried out using *Crystal Explorer 17.5* (Turner *et al.*, 2017 ▸). From the analysis of the Hirshfeld surfaces (Fig. 4 ▸), it is evident that there are differences between the chemical environments of these two identical mol­ecules. In Fig. 4 ▸, the Hirshfeld surfaces for polymorph I show a series of strong short contacts (big red dots) corresponding to hydrogen bonds stabilizing the 2D sheets. The planar areas above and below the rings are where π–π inter­actions (small red dots) take place, giving rise to the 3D network. On the other hand, polymorph II is stabilized by a short directional hydrogen bond and the sum of weak inter­actions with longer contact distances than in polymorph I. This suggests that polymorph II may be the less stable between these two phases of the title compound. To qu­anti­tatively compare polymorphs I and II in terms of their crystal packing, 2D fingerprint plots were developed and analysed. The character of the fingerprints plots for both polymorphs is similar, with small differences in the relative contributions of each type of inter­action to the Hirshfeld surface. The weak inter­actions include C⋯H (C—H⋯ π), C⋯O (C=O⋯C=O, C=O⋯π) and C⋯C (π–π), as well as short directional inter­actions such as H⋯O (Fig. 5 ▸).

**Figure 6:**
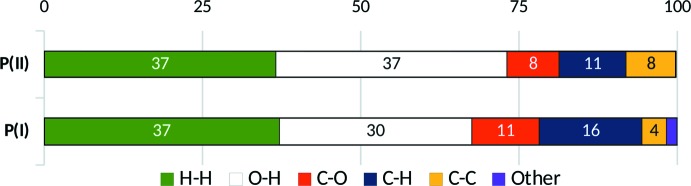
Relative contributions to the Hirshfeld surface for the major inter­molecular contacts in polymorphs I and II.

Although polymorphs I and II exhibit the same type of inter­molecular inter­actions, the way these common inter­actions contribute to the packing in each polymorph differs in each case. The minor differences in which weak inter­molecular inter­actions contribute to the formation of the crystal (Fig. 6 ▸), give rise to distinct polymorphs as suggested by Hasija & Chopra (2019 ▸). As can be seen in Fig. 6 ▸, the major forces in the crystal formation of both polymorphs are H⋯H and O⋯H inter­actions, but C⋯O and C⋯H short contacts in polymorph I make slightly bigger contributions to build the lattice, while in polymorph II, hydrogen bonding and π–π stacking contribute in greater proportions.

## Database survey   

A search of the Cambridge Structural Database (version 5.39, February 2018; Groom *et al.*, 2016 ▸) revealed only one crystal structure of the title compound (Refcode TEBFAJ; Li *et al.*, 2012 ▸). This structure, which we call polymorph I, is assembled by parallel flat sheets that extend along the *b* axis. It is worth mentioning that the acetyl coumarin without any substituent also forms at least two polymorphic forms (*A* and *B*; Munshi *et al.*, 2004 ▸) with subtle differences in inter­molecular inter­actions, which include weak C—H⋯O and C—H⋯ π inter­actions. Form *A* crystallizes with head-to-head stacking being favored during nucleation, while form *B* prefers a head-to tail-stacking. This is similar to the two polymorphs of the title compound.

## Synthesis and crystallization   

The title compound was obtained *via* Knoevenagel condensation. 3-Meth­oxy­salicyl­aldehyde and ethyl aceto­acetate in a 1:1 molar ratio were loaded in a flask with ethyl alcohol as solvent and piperidine as catalyst and left under stirring and reflux for 5 h. The product was filtered and washed with cold ethanol followed by recrystallization from ethanol to yield the title compound as colourless crystals, 88% yield, mp 444–447 K; IR νKBr (cm^−1^): 1727 (OC=O), 1682 (C=O), 1278, 1197 (C—O). NMR ^1^H (δ ppm, CDCl_3_): 8.42 (*s*, 1H, H-4); 7.25 (*d*, 1H, H-7, ^3^ *J* = 1.1, ^4^ *J* = 5.7 Hz), 7.18 (*t*, 1H, H-6, ^3^ *J* = 5.5, 2.0 Hz) 7.14 (*d*, 1H, H-5, ^3^ *J* = 2.0, ^4^ *J* = 5.7 Hz), 3.94 (*s*, 3H, OCH_3_), 2.68 (*s*, 3H, H-12). NMR ^13^C (δ ppm, CDCl_3_): 195.8 (C-11), 158.9 (C-2), 147.9 (C-4), 147.2 (C-8), 145.1 (C-9), 125.0 (C-5), 124.8 (C-3), 121.5 (C-6), 118.9 (C-10), 116.0 (C-7), 56.5 (OCH_3_), 30.8 (C-12). EA (%) calculated for C_12_H_10_O_4_: 66.05 C, 4.62 H; found: 66.15 C, 4.60 H.

## Refinement   

Crystal data, data collection and structure refinement details are summarized in Table 2 ▸. H atoms were positioned geom­etrically and treated as riding atoms, with C—H = 0.93–0.98 Å and *U*_iso_(H) = 1.2*U*_eq_(C).

## Supplementary Material

Crystal structure: contains datablock(s) I. DOI: 10.1107/S2056989019015159/dx2020sup1.cif

Supporting information file. DOI: 10.1107/S2056989019015159/dx2020Isup3.cml

Structure factors: contains datablock(s) I. DOI: 10.1107/S2056989019015159/dx2020Isup3.hkl

CCDC references: 1964910, 1964913, 1964913

Additional supporting information:  crystallographic information; 3D view; checkCIF report

## Figures and Tables

**Table 1:** Hydrogen-bond geometry (Å, °)

*D*—H⋯*A*	*D*—H	H⋯*A*	*D*⋯*A*	*D*—H⋯*A*
C12—H12*B*⋯O11^i^	0.96	2.63	3.4255 (17)	141
C6—H6⋯O2^ii^	0.93	2.45	3.3808 (16)	176
C14—H14*A*⋯O2^iii^	0.96	2.68	3.4535 (18)	138

Symmetry codes: (i) 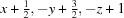; (ii) 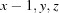; (iii) 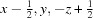.

**Table 2:** Experimental details

Crystal data	
Chemical formula	C_12_H_10_O_4_
*M*_r_	218.20
Crystal system, space group	Orthorhombic, *P* *b* *c* *a*
Temperature (K)	293
*a*, *b*, *c* (Å)	9.4973 (13), 7.9733 (11), 26.682 (4)
*V* (Å^3^)	2020.5 (5)
*Z*	8
Radiation type	Mo *K*α
μ (mm^−1^)	0.11
Crystal size (mm)	0.40 × 0.35 × 0.30 × 0.15 (radius)

Data collection	
Diffractometer	Bruker APEXII area detector
Absorption correction	For a sphere [the interpolation procedure of Dwiggins (1975 ▸) was used with some modification]
*T*_min_, *T*_max_	0.861, 0.862
No. of measured, independent and observed [*I* > 2σ(*I*)] reflections	15317, 2453, 1798
*R*_int_	0.045
(sin θ/λ)_max_ (Å^−1^)	0.667

Refinement	
*R*[*F* ^2^ > 2σ(*F* ^2^)], *wR*(*F* ^2^), *S*	0.041, 0.116, 1.10
No. of reflections	2453
No. of parameters	148
H-atom treatment	H-atom parameters constrained
Δρ_max_, Δρ_min_ (e Å^−3^)	0.20, −0.19

Computer programs: *APEX2* and *SAINT* (Bruker, 2004 ▸), *SHELXS97* and *SHELXL97* (Sheldrick, 2008 ▸), *SHELXL2018* (Sheldrick, 2015 ▸), *Mercury* (Macrae *et al.*, 2008 ▸), *WinGX2003* (Farrugia, 2012 ▸) and *PLATON* (Spek, 2009 ▸).

## supplementary crystallographic information

### Crystal data

**Table:**

C_12_H_10_O_4_	*D*_x_ = 1.435 Mg m^−^^3^
*M**_r_* = 218.20	Mo *K*α radiation, λ = 0.71073 Å
Orthorhombic, *P**b**c**a*	Cell parameters from 600 reflections
*a* = 9.4973 (13) Å	θ = 20–25°
*b* = 7.9733 (11) Å	µ = 0.11 mm^−^^1^
*c* = 26.682 (4) Å	*T* = 293 K
*V* = 2020.5 (5) Å^3^	Prism, colorless
*Z* = 8	0.40 × 0.35 × 0.30 × 0.15 (radius) mm
*F*(000) = 912	

### Data collection

**Table:**

Bruker APEXII area detector diffractometer	2453 independent reflections
Radiation source: fine-focus sealed tube	1798 reflections with *I* > 2σ(*I*)
Graphite monochromator	*R*_int_ = 0.045
φ and ω scans	θ_max_ = 28.3°, θ_min_ = 1.5°
Absorption correction: for a sphere [the interpolation procedure of Dwiggins (1975) was used with some modification]	*h* = −12→12
*T*_min_ = 0.861, *T*_max_ = 0.862	*k* = −10→10
15317 measured reflections	*l* = −34→35

### Refinement

**Table:**

Refinement on *F*^2^	Hydrogen site location: inferred from neighbouring sites
Least-squares matrix: full	H-atom parameters constrained
*R*[*F*^2^ > 2σ(*F*^2^)] = 0.041	*w* = 1/[σ^2^(*F*_o_^2^) + (0.0608*P*)^2^ + 0.0858*P*] where *P* = (*F*_o_^2^ + 2*F*_c_^2^)/3
*wR*(*F*^2^) = 0.116	(Δ/σ)_max_ < 0.001
*S* = 1.10	Δρ_max_ = 0.20 e Å^−^^3^
2453 reflections	Δρ_min_ = −0.19 e Å^−^^3^
148 parameters	Extinction correction: SHELXL2018 (Sheldrick, 2015), Fc^*^=kFc[1+0.001xFc^2^λ^3^/sin(2θ)]^-1/4^
0 restraints	Extinction coefficient: 0.0117 (15)

### Special details

**Table:**

Geometry. All esds (except the esd in the dihedral angle between two l.s. planes) are estimated using the full covariance matrix. The cell esds are taken into account individually in the estimation of esds in distances, angles and torsion angles; correlations between esds in cell parameters are only used when they are defined by crystal symmetry. An approximate (isotropic) treatment of cell esds is used for estimating esds involving l.s. planes.

### Fractional atomic coordinates and isotropic or equivalent isotropic displacement parameters (Å^2^)

**Table:**

	*x*	*y*	*z*	*U*_iso_*/*U*_eq_	
C2	0.50500 (12)	0.47624 (16)	0.37371 (4)	0.0424 (3)	
C3	0.44835 (12)	0.56358 (15)	0.41705 (4)	0.0382 (3)	
C4	0.30805 (13)	0.57565 (15)	0.42255 (4)	0.0410 (3)	
H4	0.272844	0.632762	0.450238	0.049*	
C5	0.06405 (13)	0.51543 (17)	0.39234 (5)	0.0503 (3)	
H5	0.023660	0.571130	0.419379	0.060*	
C6	−0.01876 (14)	0.44331 (19)	0.35668 (6)	0.0561 (4)	
H6	−0.116156	0.450497	0.359563	0.067*	
C7	0.03947 (14)	0.35925 (18)	0.31610 (5)	0.0512 (4)	
H7	−0.019498	0.311173	0.292263	0.061*	
C8	0.18326 (13)	0.34597 (15)	0.31060 (4)	0.0410 (3)	
C9	0.26746 (12)	0.42080 (14)	0.34702 (4)	0.0364 (3)	
C10	0.21098 (12)	0.50478 (14)	0.38783 (4)	0.0389 (3)	
C11	0.54296 (14)	0.64209 (16)	0.45541 (4)	0.0457 (3)	
C12	0.69794 (15)	0.6292 (2)	0.45047 (6)	0.0645 (4)	
H12A	0.724728	0.513243	0.449103	0.097*	
H12B	0.742035	0.681599	0.478819	0.097*	
H12C	0.727490	0.684582	0.420301	0.097*	
C14	0.17018 (17)	0.1828 (2)	0.23613 (5)	0.0637 (4)	
H14A	0.111460	0.262506	0.219101	0.096*	
H14B	0.112261	0.099779	0.252075	0.096*	
H14C	0.231370	0.129280	0.212366	0.096*	
O1	0.40976 (8)	0.40859 (11)	0.34056 (3)	0.0415 (2)	
O2	0.62587 (9)	0.45659 (16)	0.36291 (4)	0.0728 (4)	
O11	0.49013 (11)	0.71394 (14)	0.49062 (4)	0.0688 (3)	
O13	0.25265 (9)	0.26717 (11)	0.27307 (3)	0.0524 (3)	

### Atomic displacement parameters (Å^2^)

**Table:**

	*U*^11^	*U*^22^	*U*^33^	*U*^12^	*U*^13^	*U*^23^
C2	0.0323 (6)	0.0528 (8)	0.0422 (6)	−0.0022 (5)	−0.0010 (5)	−0.0078 (5)
C3	0.0384 (6)	0.0398 (7)	0.0366 (6)	−0.0002 (5)	0.0004 (5)	−0.0005 (5)
C4	0.0432 (7)	0.0407 (7)	0.0391 (6)	0.0040 (5)	0.0075 (5)	0.0005 (5)
C5	0.0355 (7)	0.0548 (8)	0.0608 (8)	0.0073 (6)	0.0079 (6)	0.0094 (6)
C6	0.0287 (6)	0.0638 (9)	0.0756 (9)	0.0034 (6)	−0.0017 (6)	0.0203 (8)
C7	0.0383 (7)	0.0542 (8)	0.0612 (8)	−0.0070 (6)	−0.0140 (6)	0.0146 (7)
C8	0.0377 (7)	0.0424 (7)	0.0428 (6)	−0.0039 (5)	−0.0060 (5)	0.0087 (5)
C9	0.0290 (6)	0.0389 (6)	0.0413 (6)	−0.0007 (5)	−0.0014 (4)	0.0072 (5)
C10	0.0341 (6)	0.0387 (7)	0.0439 (6)	0.0031 (5)	0.0041 (5)	0.0075 (5)
C11	0.0530 (8)	0.0441 (7)	0.0400 (6)	−0.0015 (6)	−0.0030 (5)	−0.0030 (5)
C12	0.0495 (8)	0.0822 (11)	0.0619 (8)	−0.0103 (7)	−0.0129 (7)	−0.0193 (8)
C14	0.0738 (10)	0.0702 (10)	0.0472 (8)	−0.0148 (8)	−0.0223 (7)	−0.0020 (7)
O1	0.0292 (4)	0.0548 (5)	0.0406 (4)	−0.0015 (4)	−0.0003 (3)	−0.0094 (4)
O2	0.0288 (5)	0.1185 (10)	0.0710 (7)	−0.0005 (5)	0.0019 (4)	−0.0424 (7)
O11	0.0716 (7)	0.0821 (8)	0.0527 (6)	0.0059 (6)	−0.0036 (5)	−0.0262 (5)
O13	0.0503 (6)	0.0613 (6)	0.0455 (5)	−0.0085 (4)	−0.0092 (4)	−0.0084 (4)

### Geometric parameters (Å, º)

**Table:**

C2—O2	1.1940 (14)	C8—O13	1.3535 (15)
C2—O1	1.3753 (14)	C8—C9	1.3929 (16)
C2—C3	1.4530 (16)	C9—O1	1.3659 (14)
C3—C4	1.3440 (16)	C9—C10	1.3863 (16)
C3—C11	1.4991 (17)	C11—O11	1.2094 (15)
C4—C10	1.4238 (17)	C11—C12	1.481 (2)
C4—H4	0.9300	C12—H12A	0.9600
C5—C6	1.362 (2)	C12—H12B	0.9600
C5—C10	1.4032 (17)	C12—H12C	0.9600
C5—H5	0.9300	C14—O13	1.4275 (15)
C6—C7	1.388 (2)	C14—H14A	0.9600
C6—H6	0.9300	C14—H14B	0.9600
C7—C8	1.3776 (17)	C14—H14C	0.9600
C7—H7	0.9300		
			
O2—C2—O1	115.19 (11)	O1—C9—C8	116.70 (10)
O2—C2—C3	127.66 (11)	C10—C9—C8	122.20 (11)
O1—C2—C3	117.15 (10)	C9—C10—C5	118.77 (11)
C4—C3—C2	119.23 (10)	C9—C10—C4	116.89 (11)
C4—C3—C11	119.32 (11)	C5—C10—C4	124.34 (11)
C2—C3—C11	121.44 (10)	O11—C11—C12	120.94 (12)
C3—C4—C10	122.85 (11)	O11—C11—C3	118.66 (12)
C3—C4—H4	118.6	C12—C11—C3	120.40 (11)
C10—C4—H4	118.6	C11—C12—H12A	109.5
C6—C5—C10	119.27 (13)	C11—C12—H12B	109.5
C6—C5—H5	120.4	H12A—C12—H12B	109.5
C10—C5—H5	120.4	C11—C12—H12C	109.5
C5—C6—C7	121.25 (12)	H12A—C12—H12C	109.5
C5—C6—H6	119.4	H12B—C12—H12C	109.5
C7—C6—H6	119.4	O13—C14—H14A	109.5
C8—C7—C6	121.00 (12)	O13—C14—H14B	109.5
C8—C7—H7	119.5	H14A—C14—H14B	109.5
C6—C7—H7	119.5	O13—C14—H14C	109.5
O13—C8—C7	126.67 (11)	H14A—C14—H14C	109.5
O13—C8—C9	115.82 (11)	H14B—C14—H14C	109.5
C7—C8—C9	117.51 (12)	C9—O1—C2	122.79 (9)
O1—C9—C10	121.09 (10)	C8—O13—C14	117.55 (11)
			
O2—C2—C3—C4	178.80 (14)	O1—C9—C10—C4	−0.53 (16)
O1—C2—C3—C4	−0.59 (17)	C8—C9—C10—C4	179.87 (11)
O2—C2—C3—C11	−0.5 (2)	C6—C5—C10—C9	0.02 (18)
O1—C2—C3—C11	−179.90 (10)	C6—C5—C10—C4	179.73 (12)
C2—C3—C4—C10	0.63 (18)	C3—C4—C10—C9	−0.08 (17)
C11—C3—C4—C10	179.96 (11)	C3—C4—C10—C5	−179.79 (11)
C10—C5—C6—C7	0.2 (2)	C4—C3—C11—O11	0.34 (18)
C5—C6—C7—C8	0.0 (2)	C2—C3—C11—O11	179.66 (12)
C6—C7—C8—O13	179.70 (11)	C4—C3—C11—C12	179.43 (12)
C6—C7—C8—C9	−0.40 (18)	C2—C3—C11—C12	−1.25 (18)
O13—C8—C9—O1	0.88 (15)	C10—C9—O1—C2	0.57 (16)
C7—C8—C9—O1	−179.03 (10)	C8—C9—O1—C2	−179.80 (11)
O13—C8—C9—C10	−179.50 (10)	O2—C2—O1—C9	−179.47 (12)
C7—C8—C9—C10	0.58 (17)	C3—C2—O1—C9	0.00 (16)
O1—C9—C10—C5	179.20 (10)	C7—C8—O13—C14	−2.70 (18)
C8—C9—C10—C5	−0.40 (17)	C9—C8—O13—C14	177.40 (11)

### Hydrogen-bond geometry (Å, º)

**Table:**

*D*—H···*A*	*D*—H	H···*A*	*D*···*A*	*D*—H···*A*
C12—H12*B*···O11^i^	0.96	2.63	3.4255 (17)	141
C6—H6···O2^ii^	0.93	2.45	3.3808 (16)	176
C14—H14*A*···O2^iii^	0.96	2.68	3.4535 (18)	138
C4—H4···O11	0.93	2.42	2.7395 (16)	100
C12—H12*A*···O2	0.96	2.52	2.797 (2)	97

Symmetry codes: (i) *x*+1/2, −*y*+3/2, −*z*+1; (ii) *x*−1, *y*, *z*; (iii) *x*−1/2, *y*, −*z*+1/2.
